# NKL homeobox gene NKX2-2 is aberrantly expressed in Hodgkin lymphoma

**DOI:** 10.18632/oncotarget.26459

**Published:** 2018-12-25

**Authors:** Stefan Nagel, Roderick A.F. MacLeod, Claudia Pommerenke, Corinna Meyer, Maren Kaufmann, Hans G. Drexler

**Affiliations:** ^1^ Department of Human and Animal Cell Lines, Leibniz Institute DSMZ-German Collection of Microorganisms and Cell Cultures, Braunschweig, Germany

**Keywords:** homeobox, NKL-code, T-ALL, Hodgkin lymphoma

## Abstract

NKL homeobox genes encode basic transcriptional regulators of cell and tissue differentiation. Recently, we described a hematopoietic NKL-code comprising nine specific NKL homeobox genes expressed in normal hematopoietic stem cells, lymphoid progenitors and during lymphopoiesis, highlighting their physiological role in the development of T-, B- and NK-cells. Here, we identified aberrant expression of the non-hematopoietic neural NKL homeobox gene NKX2-2 in about 12% of both, classical Hodgkin lymphoma (HL) and nodular lymphocyte predominant (NLP) HL patients. The NKX2-2 expressing NLPHL-derived cell line DEV served as a model by analysing chromosomal configurations and expression profiling data to reveal activating mechanisms and downstream targets of this developmental regulator. While excluding chromosomal rearrangements at the locus of NKX2-2 we identified t(3;14)(p21;q32) resulting in overexpression of the IL17 receptor gene IL17RB via juxtaposition with the IGH-locus. SiRNA-mediated knockdown experiments demonstrated that IL17RB activated NKX2-2 transcription. Overexpression of IL17RB-cofactor DAZAP2 via chromosomal gain of 12q13 and deletion of its proteasomal inhibitor SMURF2 at 17q24 supported expression of NKX2-2. IL17RB activated transcription factors FLI1 and FOXG1 which in turn mediated NKX2-2 expression. In addition, overexpressed chromatin-modulator AUTS2 contributed to NKX2-2 activation as well. Downstream analyses indicated that NKX2-2 inhibits transcription of lymphoid NKL homeobox gene MSX1 and activates expression of basic helix-loop-helix factor NEUROD1 which may disturb B-cell differentiation processes via reported interaction with TCF3/E2A. Taken together, our data reveal ectopic activation of a neural gene network in HL placing NKX2-2 at its hub, highlighting a novel oncogenic impact of NKL homeobox genes in B-cell malignancies.

## INTRODUCTION

Hodgkin lymphoma (HL) is a B-cell malignancy comprising some 10% of all lymphomas. This cancer is subclassified into two distinct entities according to clinical and histopathological features, namely classical HL (cHL) and nodular lymphocyte predominant (NLP) HL [1]. The malignant cells of HL, Hodgkin/Reed-Sternberg (HRS) cells, infiltrate lymph nodes where they are vastly outnumbered by predominant bystander cells which include activated lymphocytes, plasma cells and granulocytes [2]. This picture reflects aberrant expression of several signalling molecules by HRS cells, notably interleukins and other growth factors together with their receptors which mediate constitutive activation of the associated signalling pathways and transcription factors (TFs) [3]. TFs of the NFkB-family play a fundamental role in the pathogenesis of HL. In addition to pathway deregulation, NFkB activation occurs via genomic amplification and mutations of their negative regulators [4]. With respect to the low content of HRS cells in HL tumors, cell lines represent suitable tools to analyze the pathological mechanisms of this malignancy. While most available HL cell lines belong to the cHL group DEV represents the NLPHL entity [5, 6]. This discrimination is also supported by recent cluster analyses using expression profiling data from all bona fide HL cell lines [7].

Compromised B-cell development has been highlighted as a major aspect of the pathogenesis in HL by analyses of cell lines and micro-dissected HRS cells whose main TFs important for B-cell development are absent or inactivated, resulting in B-cells with incomplete phenotypes [8, 9]. Aberrantly downregulated B-cell associated TFs include EBF1, PAX5, POU2AF1/BOB1/OBF1 and POU2F2/OCT2 [10–13]. Additional features of disturbed B-cell differentiation in HL include repressed activity of basic helix-loop-helix (bHLH) protein TCF3/E2A by overexpressed ID2 and MSC/ABF1 [14]. However, reactivation of the fundamental TFs EBF1 or PAX5 is alone insufficient to rebuild the developmental B-cell program in HL, indicating that multiple factors are involved in complete B-cell differentiation [11, 15].

NKL homeobox genes encode TFs which regulate fundamental processes in both embryonal development and differentiation in the adult. Some represent master genes for specific cell types/organs like NKX2-3 (spleen), NKX2-5 (heart), or NKX3-1 (prostate) [16–18]. Others, like DLX family members operate a code which regulates the development of the complex structures and tissues in the embryonal region of the pharynx [19]. Accordingly, we coined the term “hematopoietic NKL-code” to describe the physiological expression pattern of particular NKL homeobox genes in early hematopoiesis and lymphopoiesis [20, 21]. We detected the physiological expression of nine NKL homeobox genes including NKX2-3 in HSCs, MSX1 in lymphoid progenitors, B-cell progenitors and NK-cells, and HHEX and NKX6-3 in late B-cell development [20–22]. Thus, due to their basic impact, aberrant activities of NKL homeobox genes may contribute to leukemogenesis/lymphomagenesis by deregulating developmental processes.

Malignant cells of T-cell acute lymphoblastic leukemia (T-ALL) are developmentally arrested thymocytes showing disturbed T-cell differentiation due to aberrant expression of particular oncogenes including NKL homeobox genes [23]. This homeobox gene subclass consists of 48 members of which 24 are aberrantly expressed in this malignancy [20, 24, 25]. Thus, NKL homeobox genes represent the largest single oncogene class in T-ALL. In B-cell malignancies deregulated expression of particular NKL homeobox genes has been also described. Thus for example, NKX2-1 is ectopically expressed in diffuse large B-cell lymphoma (DLBCL), and the hematopoietic NKL homeobox genes NKX2-3 and NKX6-3 are overexpressed while MSX1 is downregulated in particular B-cell lymphomas [21, 26–28]. Recently, we systematically analyzed deregulated NKL homeobox genes in B-cell malignancies, identifying seven overexpressed NKL-code members and six ectopically activated non-code members in patient samples including NKX2-2 in HL [21]. Collectively, these results show that NKL homeobox genes also play a significant role in B-cell neoplasms.

Here, we analyzed NKL homeobox gene NKX2-2 which is aberrantly activated in subsets of cHL and NLPHL patients. We used a NKX2-2 expressing HL cell line as a model and identified upstream regulators and downstream targets. Our data assemble a pathological gene regulatory network surrounding NKX2-2 and extend the oncogenic role of NKL homeobox genes to this type of B-cell lymphoma.

## RESULTS

### Aberrant expression of NKX2-2 in HL

**Figure 1:**
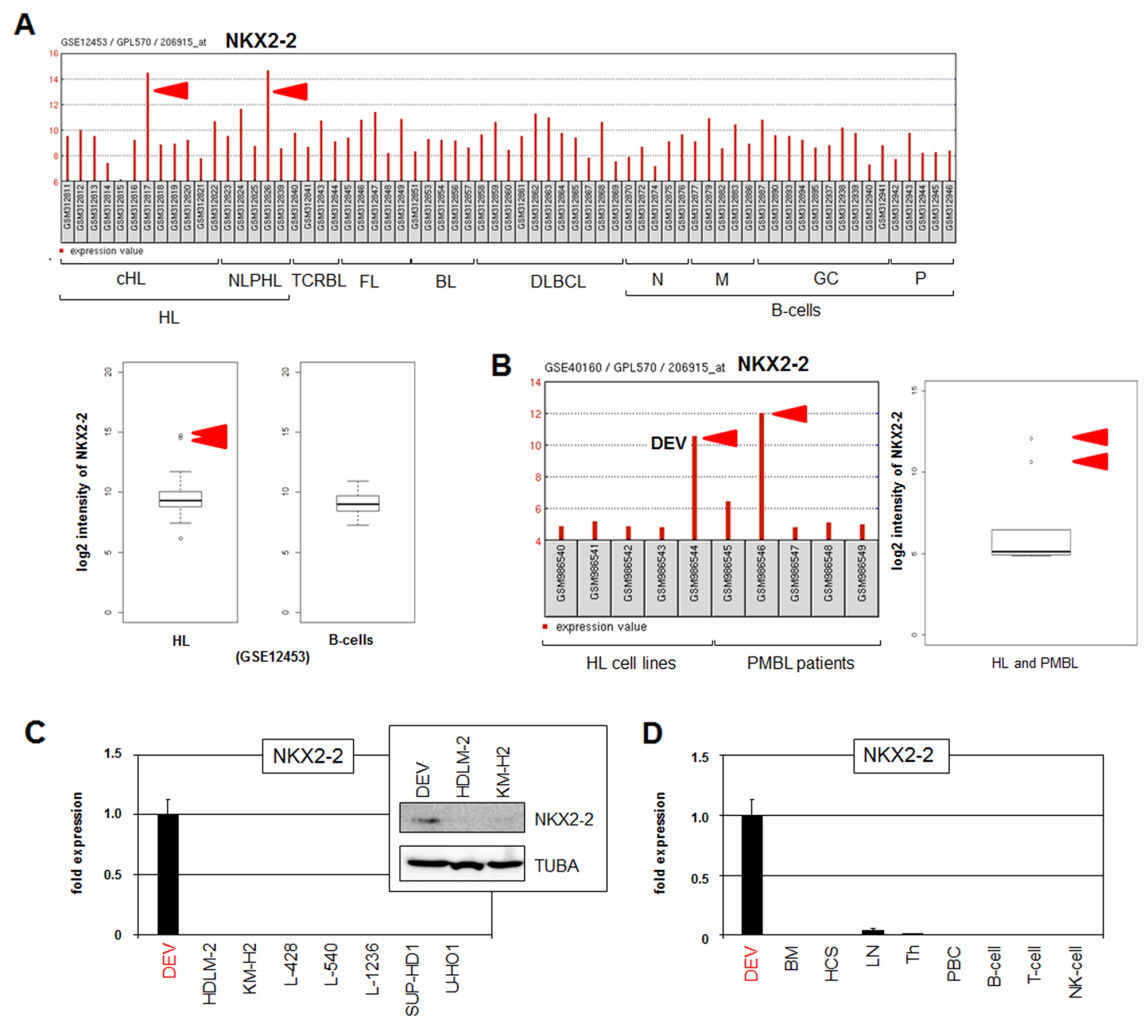
Expression of NKX2-2 in HL patients and cell lines **(A)** Expression profiling data of NKX2-2 (206915_at) in dataset GSE12453 containing samples of patients and normal B-cells are indicated as barplot (above) and boxplot (below). Hodgkin lymphoma (HL), T-cell rich B-cell lymphoma (TCRBL), follicular lymphoma (FL), Burkitt lymphoma (BL), diffuse large B-cell lymphoma (DLBCL), naïve B-cells (N), memory B-cells (M), germinal centre B-cells (GC), plasma cells (P). The red arrows indicate samples showing NKX2-2 overexpression detected in 1/12 (8%) cHL and in 1/5 (20%) NLPHL patients. **(B)** Expression profiling data of NKX2-2 in dataset GSE40160 containing samples of five HL cell lines including DEV and of five peripheral mediastinal B-cell lymphoma (PMBL) patients are indicated as barplot (left) and boxplot (right). The red arrows indicate DEV and one sample showing NKX2-2 overexpression, representing 20% of PMBL patients. **(C)** Expression analysis of NKX2-2 in HL cell lines by RQ-PCR and Western blot (insert). **(D)** RQ-PCR analysis of NKX2-2 in DEV in comparison to primary hematopoietic cell/tissue samples: bone marrow (BM), hematopoietic stem cell (HSC), lymph node (LN), thymus (Th), peripheral mononuclear blood cells (PBC), B-cells, T-cells and NK-cells.

Recently, we systematically analyzed NKL homeobox gene expression in normal B-cell development and in B-cell lymphoma patients, thereby identifying a physiological NKL-code in B-cell differentiation and aberrant activities in HL patient samples [21]. Dataset GSE12453 containes 17 HL patient samples showing overexpression of non-hematopoietic NKL homeobox gene NKX2-2 in 1 of 12 cHL and 1 of 5 NLPHL patients (Figure 1A). Examination of the additional clinical datasets GSE39134 and GSE7788 in combination with GSE12453 confirmed frequently enhanced expression of NKX2-2 in about 12% of both HL entities (Supplementary Figure 1A-1C). Of note, overexpression of this gene was detected in less than 1 % of 117 pediatric T-ALL patients supporting its significance in HL (Supplementary Figure 1D). Dataset GSE40160 contains samples of primary mediastinal B-cell lymphoma (PMBL), revealing NKX2-2 overexpression in 1 of 5 (20%) PMBL patients (Figure 1B). Therefore, frequent NKX2-2 expression in patients of the closely related B-cell malignancies HL and PMBL may support the significance of this potential oncogene in lymphomagenesis of these particular entities.

Dataset GSE40160 also contains samples from five HL cell lines (DEV, HDLM-2, KM-H2, L-428, L-1236), showing NKX2-2 overexpression in NLPHL-derived DEV (Figure 1B). Subsequent RQ-PCR analysis of eight HL cell lines confirmed NKX2-2 expression exclusively in DEV cells (Figure 1C). However, RQ-PCR analysis of four PMBL cell lines failed to detect NKX2-2 activity (Supplementary Figure 1E). Western blot analysis demonstrated NKX2-2 expression in DEV at the protein level which supported its potential functional relevance in this disease (Figure 1C). RQ-PCR analysis of NKX2-2 in DEV in comparison to primary hematopoietic cell/tissue samples showed absence in the latter (Figure 1D), indicating ectopic activity of this NKL homeobox gene in HL subsets. RNA sequencing data obtained from the human protein atlas (https://www.proteinatlas.org/) showed normal NKX2-2 expression in the brain which coincides with the first report of this gene in mice (Supplementary Figure 2) [29, 30]. Accordingly, RQ-PCR analysis of NKX2-2 in DEV and a primary human brain sample demonstrated lower but significant gene activity in the cell line and confirmed ectopic expression of this brain-associated NKL homeobox gene in HL (Supplementary Figure 3). Taken together, we demonstrated aberrant activity of NKL homeobox gene NKX2-2 in subsets of the cHL and NLPHL entities. NKX2-2 expressing NLPHL-derived cell line DEV may thus serve as useful *in vitro* model to reveal aberrant upstream factors and downstream targets of this potential oncogene in HL.

### IL17RB mediates activation of NKX2-2 in DEV

**Figure 2:**
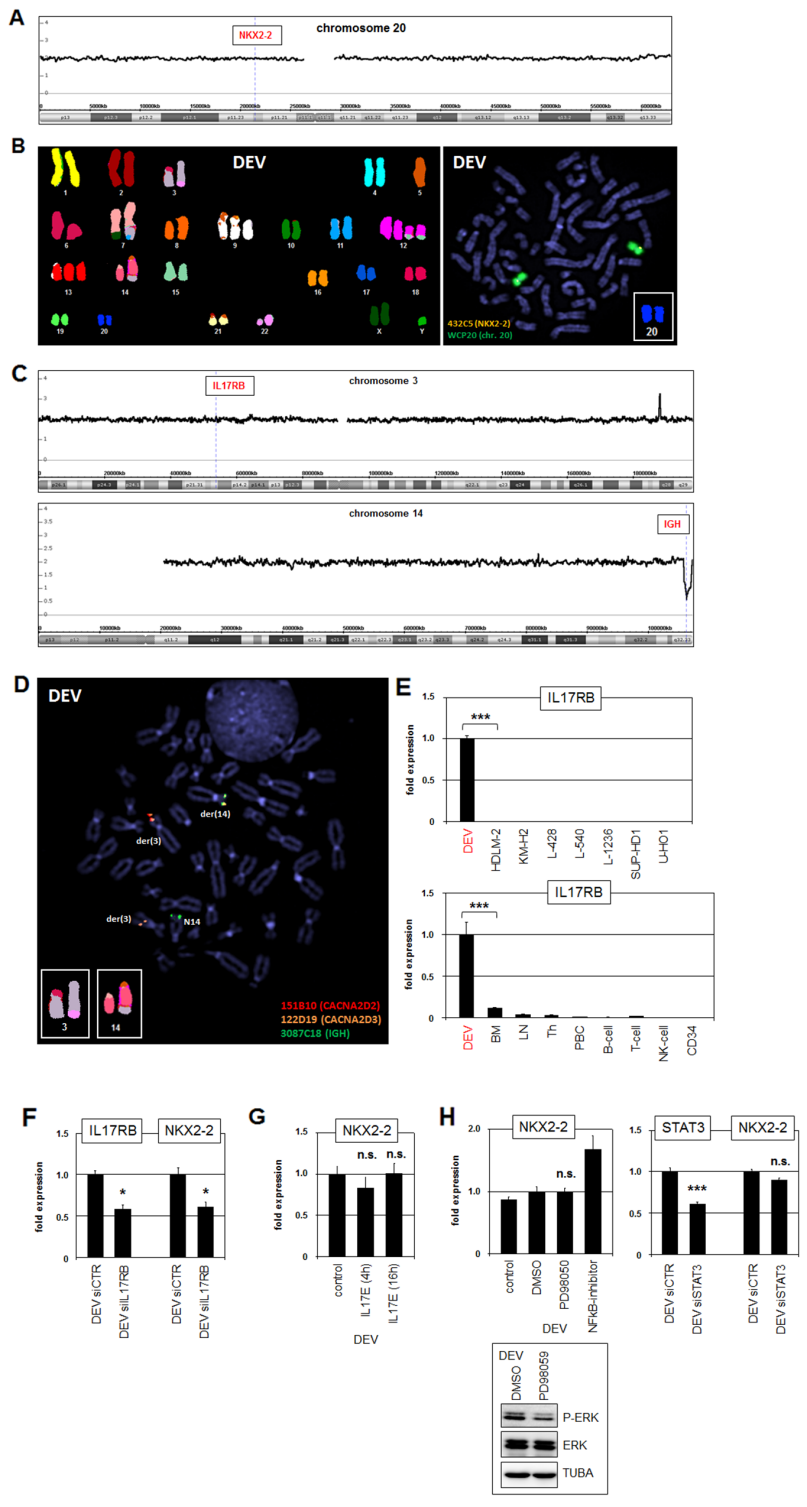
Identification of t(3;14)(p21;q32) targeting IL17RB and IGH **(A)** Genomic profiling data for chromosome 20 of HL cell line DEV. The locus of NKX2-2 is indicated showing absence of copy number gains. **(B)** SKY (left) and FISH (right) analyses of DEV. The SKY data indicate several chromosomal aberrations including t(3;14) but absence of rearrangements of NKX2-2 at 20p11. FISH was performed using whole chromosome painting probe for chromosome 20 (green) and a covering NKX2-2 specific probe (orange), indicating absence of rearrangements. **(C)** Genomic profiling data for chromosome 3 (above) and chromosome 14 (below) of HL cell line DEV. The locus of IL17RB at 3p21 is indicated showing absence of copy number gains. The IGH locus at 14q32 shows B-cell specific deletions. **(D)** FISH analysis of DEV using gene-specific probes for CACNA2D2 (red), CACNA2D3 (orange) and IGH (green). This result localized the breakpoints of t(3:14) at 3p21 between the genes CACNA2D2 and CACNA2D3 and at 14q32 nearby IGH. **(E)** RQ-PCR analyses of IL17RB in HL cell lines (above) and primary hematopoietic cell/tissue samples. **(F)** RQ-PCR analysis of IL17RB and NKX2-2 after siRNA-mediated knockdown of IL17RB. **(G)** RQ-PCR analysis of NKX2-2 after stimulation of DEV cells with recombinant IL17E/IL25 for 4h or 16h. **(H)** DEV cells were treated with ERK-inhibitor PD98059 or with NFkB-inhibitor. Subsequent analysis of NKX2-2 expression by RQ-PCR and ERK-phosphorylation by Western blot demonstrated absence of ERK-signalling or NFkB in NKX2-2 activation. RQ-PCR analysis of STAT3 and NKX2-2 after siRNA-mediated knockdown of STAT3 (left) indicated absence of STAT3-mediated NKX2-2 activation.

In T-ALL and splenic marginal zone lymphoma (SMZL) aberrant activation of particular NKL homeobox genes is conducted via chromosomal rearrangements [27, 31]. Therefore, to reveal potential genomic aberrations which may mediate deregulated expression of NKX2-2 in DEV we performed genomic profiling. However, the locus of NKX2-2 at 20p11 retained the wild type configuration and copy number gains were absent (Figure 2A). Furthermore, respective examinations by SKY and FISH using whole chromosome paints in combination with a gene-specific probe failed to detect rearrangement of the NKX2-2 locus discounting this directly operating mechanism as the cause of deregulation (Figure 2B).

The SKY data indicated additional chromosomal rearrangements including t(3;14) which might contribute, albeit indirectly, to NKX2-2 activation. Genomic profiling data showed absence of copy number alterations on the short arm of chromosome 3 and indicated a B-cell specific deletion at the rearranged IGH locus on the long arm of chromosome 14 (Figure 2C). FISH analyses narrowed one breakpoint down to region 3p21 located between the genes CACNA2D2 and CACNA2D3, and the other breakpoint to the IGH locus at 14q32 which is recurrently altered in B-cell neoplasms (Figure 2D). Analysis of expression profiling data from dataset GSE40160 for genes hosted in the indicated region at 3p21 revealed conspicuously activated IL17RB in DEV (data not shown). Subsequent RQ-PCR analysis of this gene in HL cell lines confirmed its significant activity exclusively in DEV (Figure 2E). Furthermore, expression analysis of IL17RB in DEV in comparison to primary hematopoietic cells/tissues demonstrated ectopic activation (Figure 2E). IL17RB encodes a receptor for interleukin 17 (IL17) and showed normal expression in the brain resembling NKX2-2 (Supplementary Figures 2, 3). Thus, aberrant chromosomal juxtaposition of the IGH locus to the IL17RB gene had resulted in ectopic activation of this brain-associated receptor.

To analyze the role of IL17RB in NKX2-2 expression we performed siRNA-mediated knockdown experiments. Subsequent quantification of IL17RB and NKX2-2 transcripts by RQ-PCR demonstrated concomitant downregulation, indicating that IL17RB-signalling activates NKX2-2 expression (Figure 2F). However, stimulation of DEV with its cognate recombinant ligand IL17E/IL25 showed no further increase in NKX2-2 expression levels (Figure 2G), suggesting maximal activity of this receptor in DEV cells. Aberrant downstream effects of IL17RB include promotion of ERK-signalling in thyroid cancer cells, and activation of NFkB or STAT3 in breast cancer cells [32–34]. However, although treatment of DEV with ERK-inhibitor PD98059 reduced phosphorylation of ERK, NKX2-2 expression levels remained unaltered (Figure 2H). Similarly, treatment of DEV with NFkB-inhibitor or by siRNA-mediated knockdown of STAT3 showed even activating or no effect on NKX2-2 expression, respectively (Figure 2H). Taken together, we identified a novel chromosomal aberration in HL cell line DEV, t(3;14)(p21;q32), which activates IL17RB expression via juxtaposition to the IGH locus. Furthermore, this receptor mediated NKX2-2 activation without involvement of ERK-signalling, NFkB or STAT3.

### DAZAP2 and SMURF2 regulate NKX2-2 expression

**Figure 3:**
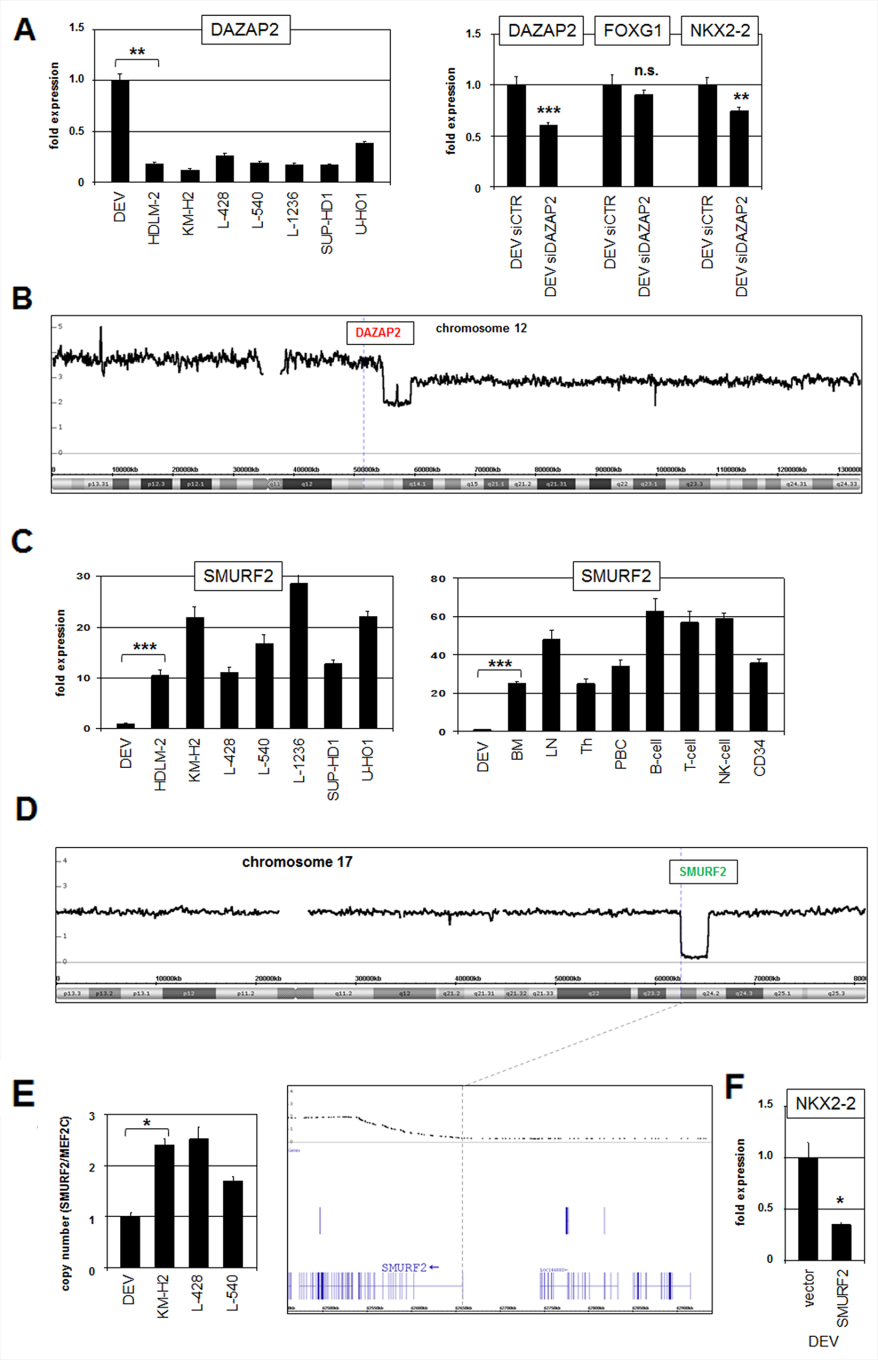
DAZAP2 and SMURF2 are deregulated modulators of IL17RB activity **(A)** RQ-PCR analysis of DAZAP2 in HL cell lines (left). RQ-PCR analysis of DAZAP2, FOXG1 and NKX2-2 in DEV cells treated for siRNA-mediated knockdown of DAZAP2 (right). **(B)** Genomic profiling data for chromosome 12 of HL cell line DEV. The locus of DAZAP2 is indicated and located in a region of copy number gain. **(C)** RQ-PCR analysis of SMURF2 in HL cell lines (left) and in primary hematopoietic cell/tissue samples (right). **(D)** Genomic profiling data for chromosome 17 of HL cell line DEV. The locus of SMURF2 is indicated and located in deleted region. Enlargement of this deleted region demonstrates that the promoter region and the 5’-primed end of SMURF2 is lost (below). **(E)** Quantification of exon 1 of SMURF2 in genomic DNA of selected HL cell lines by Q-PCR. The gene MEF2C served as reference. **(F)** RQ-PCR analysis of NKX2-2 in DEV cells treated with forced expression of SMURF2.

The activity of IL17RB is supported by the cofactor DAZAP2 which in turn is regulated via proteasomal degradation mediated by SMURF2 [35]. RQ-PCR analysis of DAZAP2 demonstrated enhanced expression levels in DEV (Figure 3A). SiRNA-mediated knockdown of DAZAP2 resulted in reduced transcription of NKX2-2 (Figure 3A), confirming the activating role of DAZAP2 described for IL17RB. Genomic profiling data for DAZAP2 at 12q13 showed a copy number gain of its locus due to a duplication of extended parts of chromosome 12 which may underlie its enhanced gene activity in DEV (Figure 3B).

The expression level of SMURF2 was strongly reduced in DEV as compared to HL control cell lines and primary hematopoietic cell/tissue samples (Figure 3C). Interestingly, copy number data of DEV demonstrated a site-directed genomic deletion of SMURF2 at 17q23 (Figure 3D). Quantification of genomic exon 1 copies from SMURF2 by RQ-PCR confirmed the indicated loss of this gene and its regulatory upstream region (Figure 3E). Forced expression of SMURF2 in DEV resulted in reduced NKX2-2 expression (Figure 3F), confirming the activating role of IL17RB and its negative control by SMURF2. Thus, elevated DAZAP2 and reduced SMURF2 gene activities were effected by chromosomal aberrations and contributed to increased NKX2-2 transcription probably via elevated activity of IL17RB.

### Aberrant activity of neuronal TFs in DEV

**Figure 4:**
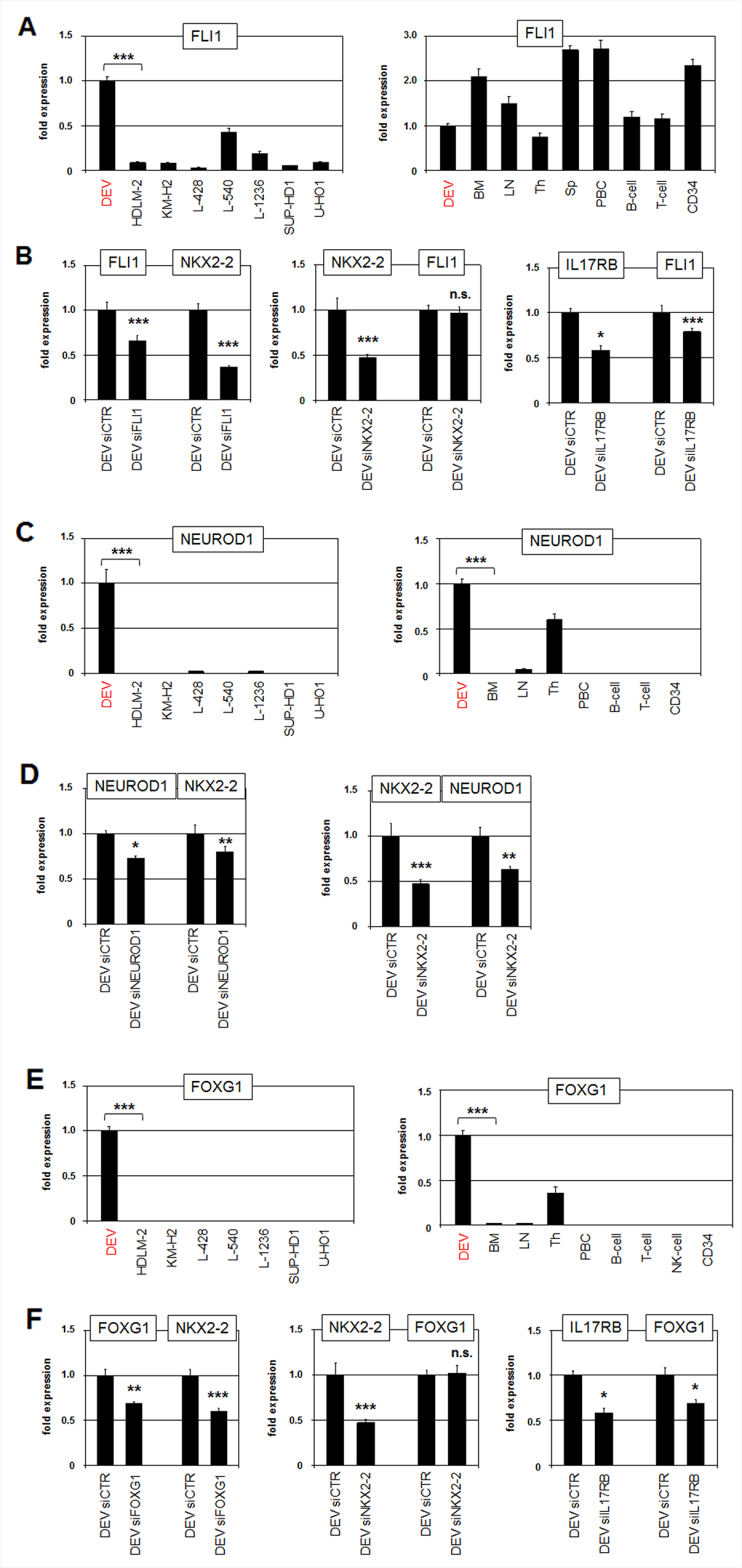
Neuronal TFs FLI1, NEUROD1 and FOXG1 activate NKX2-2 expression **(A)** RQ-PCR analysis of FLI1 in HL cell lines (left) and primary hematopoietic cell/tissue samples (right). **(B)** RQ-PCR analysis of FLI1 and NKX2-2 in DEV cells after siRNA-mediated knockdown of FLI1 (left). RQ-PCR analysis of NKX2-2 and FLI1 in DEV cells after siRNA-mediated knockdown of NKX2-2 (middle). RQ-PCR analysis of IL17RB and NKX2-2 in DEV cells after siRNA-mediated knockdown of IL17RB (right). **(C)** RQ-PCR analysis of NEUROD1 in HL cell lines (left) and primary hematopoietic cell/tissue samples (right). **(D)** RQ-PCR analysis of NEUROD1 and NKX2-2 in DEV cells after siRNA-mediated knockdown of NEUROD1 (left). RQ-PCR analysis of NKX2-2 and NEUROD1 in DEV cells after siRNA-mediated knockdown of NKX2-2 (right). **(E)** RQ-PCR analysis of FOXG1 in HL cell lines (left) and primary hematopoietic cell/tissue samples (right). **(F)** RQ-PCR analysis of FOXG1 and NKX2-2 in DEV cells after siRNA-mediated knockdown of FOXG1 (left). RQ-PCR analysis of NKX2-2 and FOXG1 in DEV cells after siRNA-mediated knockdown of NKX2-2 (middle). RQ-PCR analysis of IL17RB and FOXG1 in DEV cells after siRNA-mediated knockdown of IL17RB (right).

NKX2-2 is aberrantly expressed in Ewing sarcoma. In this malignancy the TF encoding gene FLI1 is fused to EWS and the resultant hybrid protein directly activates NKX2-2 transcription [36]. To examine if FLI1 plays a role in NKX2-2 expression in DEV as well, we first quantified FLI1 transcripts in HL cell lines and primary hematopoietic cells/tissues (Figure 4A). DEV showed elevated transcript levels as compared to HL control cell lines. FLI1 transcripts were detected in all analyzed hematopoietic samples of which spleen expressed the highest levels. Public RNA-sequencing data showed high FLI1 expression levels in the spleen and lower levels in the remaining tissues including the brain (Supplementary Figure 2). The expression levels of FLI1 were similar in DEV and the brain sample as analyzed by RQ-PCR (Supplementary Figure 3). SiRNA-mediated knockdown of FLI1 in DEV resulted in concomitantly reduced expression of NKX2-2, indicating an activating input for FLI1 in HL (Figure 4B). SiRNA-mediated knockdown of NKX2-2 showed no effect on FLI1 expression, discounting mutual regulation (Figure 4B). In contrast, knockdown of IL17RB resulted in decreased FLI1 expression, demonstrating that NKX2-2 activator FLI1 is located downstream of the IL17-receptor IL17RB (Figure 4B).

Physiologically, NKX2-2 operates in the development of the brain and additionally in the pancreas [30, 37]. In the latter context, NKX2-2 has been shown to regulate NEUROD1 directly [38, 39]. Of note, this bHLH TF is also involved in neuronal differentiation indicating that its regulatory connection with NKX2-2 may be more widespread [40]. RQ-PCR analysis and RNA-seq data of NEUROD1 transcripts in HL cell lines and primary cells/tissues detected significant expression in DEV, in the thymus and the brain (Figure 4C, Supplementary Figures 2, 3). SiRNA-mediated knockdown of NEUROD1 and NKX2-2 demonstrated mutual activation of these genes (Figure 4D), supporting that NEUROD1 represents a downstream target which also plays a role in NKX2-2 deregulation in HL.

To identify additional activators of NKX2-2 we performed comparative expression profiling analysis of dataset GSE40160 using R-based online tools. This examination revealed 250 significant differentially expressed genes in DEV as compared to the four associated HL control cell lines, including IL17RB, SYK, INPP5D, FOXG1 and AUTS2 (Supplementary Figure 4). Of note, the identification of IL17RB confirmed our data as described above. Furthermore, elevated expression of B-cell receptor signalling components SYK and INPP5D highlight the NLPHL character of DEV while the controls represent the cHL entity [5, 7, 41]. FOXG1 encodes a TF of the forkhead-family and is involved in the development of the brain and the thymus [42, 43]. Consistently, RQ-PCR analysis of FOXG1 demonstrated exclusive activity in HL cell line DEV and in the thymic and brain samples (Figure 4E, Supplementary Figures 2, 3). SiRNA-mediated knockdown of FOXG1 in DEV resulted in concomitantly reduced expression of NKX2-2, indicating an activating role of this TF (Figure 4F). SiRNA-mediated knockdown of NKX2-2 showed no effect on FOXG1 transcription, discounting mutual regulation (Figure 4F). Furthermore, knockdown of IL17RB resulted in decreased FOXG1 expression, demonstrating that this NKX2-2 activator is located downstream of IL17RB as well (Figure 4F).

Public RNA-sequencing data showed overlapping expression patterns of NKX2-2, IL17RB, FLI1, NEUROD1 and FOXG1 in the brain (Supplementary Figure 2), suggesting regulatory relationships in this tissue and aberrant reactivation of this network in HL. Thus, we identified three neuronal TFs which are involved in NKX2-2 expression in DEV. FLI1 and FOXG1 are located downstream of IL17RB while NEUROD1 and NKX2-2 activate each other.

### AUTS2 and MSX1 regulate NKX2-2 in DEV

**Figure 5:**
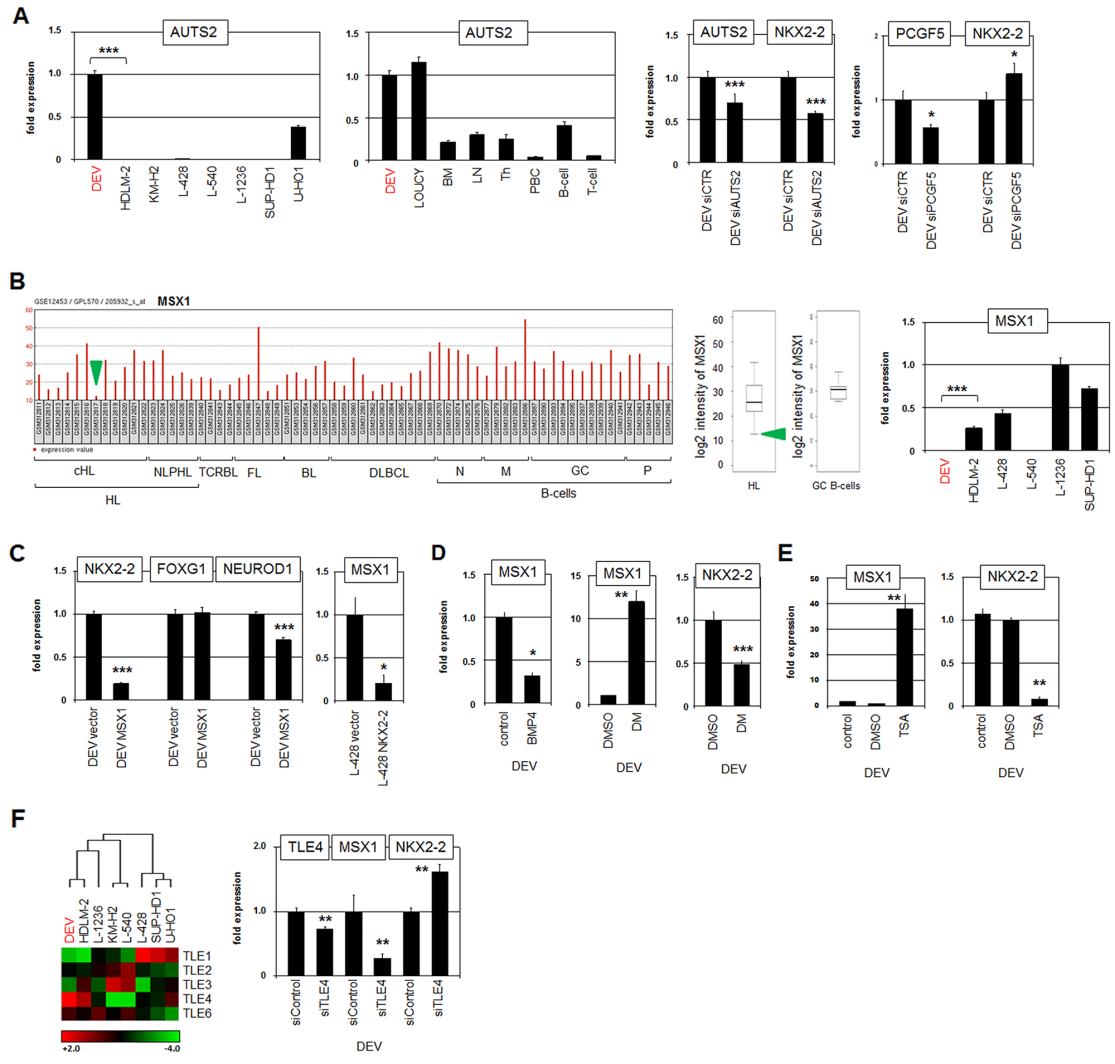
AUTS2, MSX1 and TLE4 regulate NKX2-2 expression **(A)** RQ-PCR analysis of AUTS2 in HL cell lines (left) and primary hematopoietic cell/tissue samples (middle). RQ-PCR analysis of AUTS2 and NKX2-2 in DEV cells after siRNA-mediated knockdown of AUTS2 (middle). RQ-PCR analysis of PCGF5 and NKX2-2 in DEV cells after siRNA-mediated knockdown of PCGF5 (right). **(B)** Expression profiling data of MSX1 (205932_s_at) in dataset GSE12453 containing samples of patients and normal B-cells are indicated as barplot (left) and boxplot (middle). Hodgkin lymphoma (HL), T-cell rich B-cell lymphoma (TCRBL), follicular lymphoma (FL), diffuse large B-cell lymphoma (DLBCL), naïve B-cells (N), memory B-cells (M), germinal centre B-cells (GC), plasma cells (P). The green arrows indicate HL patients showing reduced expression of MSX1. RQ-PCR analysis of MSX1 in HL cell lines (left). **(C)** RQ-PCR analysis of NKX2-2, FOXG1 and NEUROD1 in DEV cells treated for forced expression of MSX1 (left). RQ-PCR analysis of MSX1 in L-428 cells treated for forced expression of NKX2-2 (left). **(D)** RQ-PCR analysis of MSX1 and NKX2-2 in DEV cells stimulated with BMP4 or BMP-signalling inhibitor dorsomorphin (DM). **(E)** RQ-PCR analysis of MSX1 (left) and NKX2-2 (right) in DEV cells treated with HDAC-inhibitor TSA. **(F)** Heat map showing gene expression values of TLE genes in HL cell lines (left), indicating elevated expression of TLE4 in DEV. Red indicates high, black medium and green low expression levels. RQ-PCR analysis of TLE4, MSX1 and NKX2-2 in DEV cells after siRNA-mediated knockdown of TLE4 (right).

Additionally, our comparative expression profiling approach revealed significantly enhanced activity of AUTS2 in DEV (Supplementary Figure 4). AUTS2 encodes a chromatin regulator, interacts with polycomb protein PCGF5, and converts the associated repressor complex into an activator [44]. RQ-PCR analysis of AUTS2 in HL cell lines and primary hematopoietic cell/tissue samples confirmed elevated expression levels in DEV (Figure 5A). Interestingly, DEV expressed similar AUTS2 levels as T-ALL cell line LOUCY in which this regulator is overexpressed driving activation of NKL homeobox gene MSX1 [43]. SiRNA-mediated knockdown of AUTS2 in DEV resulted in reduced expression levels of NKX2-2 (Figure 5A), indicating that AUTS2 also activates NKL homeobox gene NKX2-2. Consistently, siRNA-mediated knockdown of PCGF5 resulted in slightly increased expression of NKX2-2 (Figure 5A), confirming the regulatory interplay between AUTS2 and PCGF5 in HL cells.

NKL homeobox gene MSX1 is downregulated in several HL cell lines [28]. Reduced MSX1 levels were consistently detected in HL patient subsets and additionally in DEV (Figure 5B). To analyze regulatory connections between MSX1 and NKX2-2 we overexpressed MSX1 in DEV. This treatment resulted in reduced expression of NKX2-2 and its target NEUROD1 but left FOXG1 unperturbed (Figure 5C). Forced expression of NKX2-2 in HL cell line L-428 resulted in reduced transcript levels of MSX1 (Figure 5C), indicating mutual repression of these NKL homeobox genes. In T-ALL cell line LOUCY, MSX1 is additionally regulated by repressive BMP-signalling and by activating histone acetylation [45, 46]. Treatment of DEV with recombinant BMP4 or BMP receptor inhibitor dorsomorphin yielded consistent results in this HL cell line (Figure 5D). Due to the repressive impact of MSX1, dorsomorphin treatment resulted in increased MSX1 and decreased NKX2-2 expression (Figure 5D). In the same way, treatment with histone deacetylase inhibitor TSA resulted in increased MSX1 and decreased NKX2-2 expression as well (Figure 5E).

NKL homeodomain proteins interact with corepressors of the TLE/Groucho family via the conserved EH1-motif in their N-terminal region [47]. Expression profiling analysis of TLE family members showed differences between HL cell lines with DEV expressing elevated levels of TLE4 (Figure 5F). Subsequent siRNA-mediated knockdown of TLE4 in DEV resulted in reduced expression of MSX1 and elevated expression of NKX2-2 (Figure 5F). These data may indicate that MSX1 (or another factor) recruited corepressor TLE4 to repress NKX2-2. Thus, constraining the repressive potential of the acting repressor resulted in increased expression of NKX2-2 which in turn inhibits MSX1 transcription. Taken together, these results show a tightly controlled network consisting of NKL homeobox genes, chromatin regulators and corepressors. Of note, comparative expression profiling of selected cHL patient samples indicated significant differences in gene activities of NKX2-2, FOXG1, NEUROD1 and SMURF2 (Supplementary Figure 5), demonstrating that parts of the described network in NLPHL-derived DEV are relevant in both NLPHL and cHL entities.

### Functional analysis of NKX2-2 in HL

Finally, to uncover potential oncogenic functions of aberrant NKL homeobox gene NKX2-2 activities in HL we analyzed selected public expression profiling datasets using R-based online tools. We compared NKX2-2 expressing DEV with four control cell lines (GSE40160, Supplementary Table 1), four cHL patients expressing high NKX2-2 levels with four negative controls (GSE39134, Supplementary Table 2), and one NLPHL patient expressing high levels of NKX2-2 with six negative controls (GSE7788, Supplementary Table 3). The top 250 most significant differentially expressed genes were subsequently used for gene set annotation analyses. However, the obtained KEGG-pathways and GO-terms indicated no common pathway or mechanism typically deregulated in cancer cells including apoptosis and cell cycle (not shown). Accordingly, DEV cells treated by siRNA-mediated knockdown of NKX2-2 showed after three days no significant difference to controls concerning cell growth and survival as analyzed microscopically (data not shown). Additional treatment with etoposide showed also no difference in apoptosis as analyzed by caspase-assay and MTT assay (Supplementary Figure 6). Thus, these data demonstrated the lack of functional impacts in survival or proliferation by aberrantly expressed NKL homeobox gene NKX2-2 in HL.

## DISCUSSION

**Figure 6:**
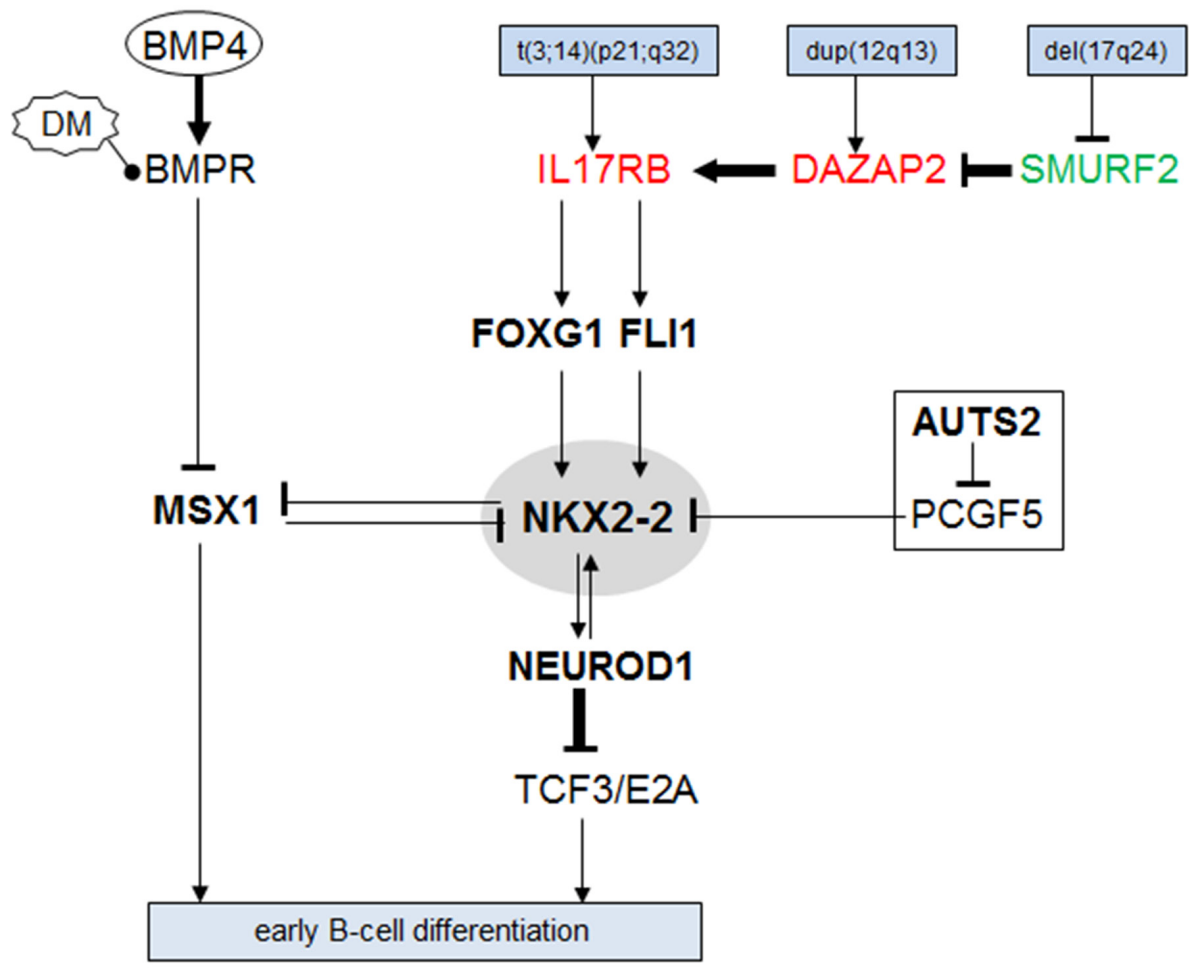
Aberrant gene regulatory network around NKX2-2 This diagram summarizes the results of this study as a gene regulatory network. NKX2-2 is located in the center and is highlighted by a grey oval. IL17RB is activated by t(3;14)(p21;q32) and elevated DAZAP2 and reduced SMURF2. The TFs FOXG1 and FLI1 are located downstream of IL17RB and mediated NKX2-2 activation. MSX1 is regulated by the BMP-pathway and represents a mutual repressor of NKX2-2. Chromatin-modulator AUTS2 transforms repressor PCGF5 into an activator of NKX2-2. NEUROD1 is capable to interact and deregulate with TCF3 which represents an important regulator of B-cell differentiation deregulated in HL [14, 46].

A summary of our study is depicted in Figure 6. In cHL and NLPHL patients as well as in NLPHL-derived cell line DEV we detected aberrant expression of NKL homeobox gene NKX2-2. After excluding genomic and chromosomal rearrangements at the NKX2-2 locus in DEV we identified a novel chromosomal aberration, t(3;14)(p21;q32), which activates IL17RB expression via juxtaposition to the IGH locus. At the same time the genes DAZAP2 and SMURF2 were also deregulated via genomic aberrations and supported NKX2-2 expression probably via co-activation of IL17RB [35]. Downstream of IL17RB we identified the TFs FOXG1 and FLI1 which in turn activate NKX2-2 transcription. Moreover, B-cell progenitor associated NKL homeobox gene MSX1 is suppressed in HL subsets, regulated by the BMP-pathway and acts as a mutual repressor of NKX2-2. Finally, the overexpressed chromatin-regulator AUTS2 contributed to NKX2-2 expression probably via conversion of the repressor PCGF5 into an activator [44]. Downstream of NKX2-2 we identified the mutual activator NEUROD1. This bHLH protein has been shown to interact with TCF3/E2A playing an important role in B-cell differentiation [14, 48]. We failed to detect a role of NKX2-2 in apoptosis or proliferation supporting that this aberrantly expressed NKL homeobox gene primarily disturbed B-cell differentiation via deregulation of developmental genes including MSX1 and TCF3.

NKX2-2 is normally expressed in the developing brain and pancreas [30, 37, 49], where several members of the aberrant gene network described here are also expressed and involved in the embryonal development of these organs [39, 40, 43, 48, 50]. RNA-sequencing data obtained from the human protein atlas demonstrated the expression of NKX2-2, IL17RB, FLI1, FOXG1 and NEUROD1 in the brain [29]. Therefore, our data indicate that regulatory relationships normally active in neuronal and pancreatic differentiation are aberrantly activated in HL and deregulate the differentiation of B-cells.

Sonic hedgehog (SHH)-signalling activates NKX2-2 expression in the ventral neural tube development [50, 51]. However, components of the SHH-signalling pathway were not detected in our comparative expression profiling approaches of DEV and HL patient samples using gene set annotation enrichment analysis (Supplementary Tables 1-3), discounting an activating role for SHH. NKX2-2 is part of a homeodomain protein code which controls the differentiation of the ventral neural tube. Code members are activated or repressed by SHH-signalling and show cross-regulatory activity [51]. Recently, we established an NKL-code in B-cell development the members of which also generate a gene regulatory network by mutual regulation [21]. In this study, we have documented that BMP-signalling impacts the activity of NKL-code member MSX1 and its cross-regulatory connection with NKX2-2, demonstrating structural similarities in the generation of homeobox gene-codes in different tissues.

NKL homeobox gene NKX2-2 is aberrantly expressed in T-ALL as well [52]. In this lymphatic malignancy NKX2-2 is directly activated via chromosomal rearrangement t(14;20)(q11;p11) after juxtaposition with the T-cell receptor locus. However, in T-ALL NKX2-2 is expressed in less than 1% of pediatric patients, thus, representing a rare oncogene of the NKL homeobox gene subclass in this malignancy. Our data indicate NKX2-2 expression in about 12% of HL patients resembling the (usually chromosomal) activation frequency of NKL homeo-oncogene TLX1 in adult T-ALL [23]. In HL, chromosomal rearrangements are frequent and involve chromothripsis, explaining the singular dearth of recurrent aberrations in this entity [53–59]. Accordingly, our SKY data for DEV revealed multiple chromosomal abnormalities.

Here, we identified and characterized a novel non-recurrent aberration in DEV, t(3;14)(p21;q32), which activates IL17RB. Our data also demonstrated normal IL17RB expression in the brain. However, several reports show that this receptor gene plays a role in T-cell development. Accordingly, IL17RB is expressed in invariant NK/T-cells and in double negative developing T-cells where it is regulated by GATA3 [60–62]. Of note, ETS-family factor FLI1 is activated by IL17RB as shown here and is expressed in early T-cell and NK-cell development which may suggest that this regulatory relationship also plays a role in these cells [63–65]. In HTLV-positive adult T-cell leukemia IL17RB is overexpressed as well indicating an oncogenic function in this disease [66]. Other reports show that IL17RB is normally expressed in decidual stroma cells and plays a role in breast cancer [67–69]. Thus, in the hematopoietic system IL17RB is expressed in the T- and NK-cell lineages but not in B-cells, highlighting its ectopic activity in HL. The NLPHL-derived cell line DEV contains in addition to the IL17RB targeting t(3;14)(p21;q32) a chromosomal duplication activating DAZAP2 at 12q13, and a genomic deletion targeting SMURF2 at 17q23. Analyses of copy number alterations from 19 NLPHL patients show chromosomal gains at 3p and 12q and losses of chromosome 17 [70]. These data correspond with our findings and support that IL17RB-signalling may represent an aberrantly activated pathway in NLPHL.

Normal expression of NKX2-2 is additionally regulated via chromatin-modifications. Bivalent chromatin contains both activating and repressive histone-marks and is established in embryonal stem cells (ESCs) at key developmental genes including NKX2-2 [71]. This mark remains at the NKX2-2 locus during differentiation of ESCs to hemangioblasts [72]. NKX2-2 is also regulated during ESC-differentiation by polycomb repressor complex (PRC)1 [73]. Our data show that PCGF5 and AUTS2 are involved in NKX2-2 (de)regulation. AUTS2 interacts with and transforms PCGF5 - a component of repressor complex subtype PRC1.5 [45]. AUTS2 plays a physiological role in NK-cell and early B-cell development [43]. Overexpression of AUTS2 drives aberrant activation of NKL homeobox gene MSX1 in T-ALL [52], highlighting the importance of this chromatin-regulator in both normal and malignant lymphopoiesis.

Deregulations of bHLH factors play a fundamental role in the development of HL [14]. Interaction of the NKX2-2 target NEUROD1 with TCF3 may represent an additional example for this pathological mechanism [48]. NKX2-2 itself is also able to interact with particular bHLH proteins, however, it does not bind to NEUROD1 [74]. Common protein binding partners of the NKL homeodomain proteins are corepressors of the Groucho/TLE family [47]. Therefore, interactions with TLEs or histone H1 may underlie their oncogenic activity as suggested previously for T-ALL and HL, respectively [28, 75]. Furthermore, TCF4 interacts with corepressor TLE4 thereby repressing NKX2-2 [76, 77]. MSX1 suppresses NKX2-2 expression as shown in this study indicating that TLE-corepressors play also a role in HL [78]. Consistently, TLE4 has been shown to act as a corepressor of oncogenic homeodomain protein SIX1 in HL, mediating suppression of B-cell factor SPIB [79]. Suppression of SPIB in HL is also performed by aberrantly expressed NKL homeodomain protein HLX [80], highlighting the impact of NKL homeobox genes in deregulation of B-cell differentiation.

In conclusion, we identified aberrant expression of NKL homeobox gene NKX2-2 in HL which supports the oncogenic role of this gene subclass in lymphoid malignancies. Their widespread activity in leukemia and lymphoma indicates a common function of this group of homeobox genes which might hence represent a promising target for therapies. Their particular expression patterns may serve as diagnostic markers as well. In addition, our data document the fitness of DEV to model NLPHL at the molecular level, the only cell line hitherto available for investigating this entity in the laboratory.

## MATERIALS AND METHODS

### Expression profiling and bioinformatics

Public expression profiling datasets of lymphoma/leukemia patients were obtained from Gene Expression Omnibus (GEO; https://www.ncbi.nlm.nih.gov/gds): GSE12453 (containing samples of 17 HL patients, of 25 B-Non-Hodgkin lymphoma and of 25 normal B-cells) [9], GSE39134 (29 cHL samples) [81], GSE7788 (10 NLPHL samples) [82], GSE40160 (samples from five HL cell lines and five primary mediastinal B-cell lymphoma patients) [40], and GSE26713 (117 T-ALL samples) [52]. These gene expression microarray profiling data were generated using the HG U133 Plus 2.0 gene chip (Affymetrix, High Wycombe, UK). Expression values were given as barplots obtained directly from the website or as boxplots using R-packages (https://www.bioconductor.org/). Statistical significance was calculated using online tools provided by GEO, or using R-based Students *T*-Test. The obtained p-values are indicated.

Expression profiling datasets of seven HL cell lines using HG U133 Plus 2.0 gene chip (Affymetrix) were generated by Prof. Andreas Rosenwald (Institute of Pathology, University of Würzburg, Germany) and by Dr. Robert Geffers (Genome Analytics, Helmholtz Centre for Infection Research, Braunschweig, Germany). The datasets are available at GEO (GSE115191). Expression profiling data for HL cell line DEV were obtained from dataset GSE40160. After RMA-background correction and quantile normalization of the spot intensities, the profiling data were expressed as ratios of the sample mean and subsequently log2 transformed. Data processing was performed via R/Bioconductor using limma and affy packages. For creation of heat maps we used CLUSTER version 2.11 and TREEVIEW version 1.60 (http://derisilab.ucsf.edu/microarray/software/ClusterTreeView.pdf). To reveal potential biological function of shortlisted genes, gene-annotation enrichment analysis was performed using DAVID bioinformatics resources [83].

### Cell lines and treatments

HL-cell lines HDLM-2, KM-H2, L-428, L-540, L-1236, SUP-HD1, U-HO1 and PMBL cell line U-2940 are held by the DSMZ (Braunschweig, Germany) and were cultivated as described [84]. HL cell line DEV was kindly provided by Dr. Van den Berg (University of Groningen, NL). PMBL cell line FARAGE, KARPAS-1106P and MED-B1 were obtained from the original investigators [85]. Cell stimulations were performed for 16 h by treatment with 20 ng/ml recombinant human protein IL17E (R&D Systems, Wiesbaden, Germany), with 14 μM NFkB-activation-inhibitor (EMD Millipore, Darmstadt, Germany), with 10 μg/ml trichostatin A (TSA, Sigma, Taufkirchen, Germany), with 35 μM PD98059 (Sigma), and with 100 μM etoposide (Sigma). Gene specific siRNA oligonucleotides and AllStars negative Control siRNA (siControl) were obtained from Qiagen (Hilden, Germany). Expression constructs for NKX2-2, MSX1 and SMURF2 were obtained from Origene (Wiesbaden, Germany). SiRNAs (80 pmol) and expression constructs/vector controls (2 μg) were transfected into 1x10^6^ cells by electroporation using the EPI-2500 impulse generator (Fischer, Heidelberg, Germany) at 350 V for 10 ms. Transfected cells were harvested after 20 h cultivation. For functional examinations treated DEV cells were stained by trypan blue (Sigma) and counted microscopically (Neubauer chamber) or analysed by the IncuCyte S3 Live-Cell Analysis System (Essen Bioscience, Hertfordshire, UK). For detection of apoptotic cells we used the IncuCyte Caspase-3/7 Green Apoptosis Assay (Essen Bioscience).

### MTT assay

DEV cells were transfected as indicated and and treated for 20 h with 100 μM Etoposide (Sigma) which has been dissolved in dimethylsulfoxide, and subsequently prepared for standardized MTT (3-(4,5-dimethylthiazol-2-yl)-2,5-diphenyltetrazolium bromide; obtained from Sigma) assays. The measurement was performed twice in triplicates. The absorbance was determined at 540 nm and at 620 nm as background control using ELISA reader Multiskan EX (Thermo Electron, Vantaa, Finland).

### Genomic and chromosomal analyses

For genomic profiling genomic HL-cell line DNA was prepared using the Qiagen Gentra Puregene Kit (Qiagen). Labelling, hybridization and scanning of HD Cytoscan arrays were performed at the Genome Analytics Facility, Helmholtz Centre for Infection Research (Braunschweig, Germany), according to the manufacturer’s protocols (Affymetrix). Data were interpreted using the Chromosome Analysis Suite software version 2.0.1.2 (Affymetrix).

Chromosomal analysis by Spectral Karyotyping (SKY) and fluorescence in situ hybridization (FISH) was performed as described previously [86]. SKY probes and whole chromosome painting probes were obtained from Applied Spectral Imaging (Neckarhausen, Germany). BAC clones were obtained from BacPac Resources, Children’s Hospital Oakland Research Institute (CA, USA) to analyze NKX2-2 (RP11-151B10, RP11-122D19, RP11-308C18) and IGH (RP11-3087C18). Insert DNA was harvested using the Big BAC DNA Kit (Princeton Separations, Adelphia, NJ, USA) and directly labelled by nick translation with dUTP-fluors (Dyomics, Jena, Germany). Fluorescent images were captured and analyzed with an Axio-Imager microscope (Zeiss, Göttingen, Germany) configured to a dual Spectral Imaging FISH system (Applied Spectral Imaging).

### Polymerase chain-reaction (PCR) analyses

Total RNA was extracted from cell line samples using TRIzol reagent (Invitrogen, Darmstadt, Germany). Primary human total RNA used in this study was commercially obtained - isolated from peripheral blood mononuclear cells (PBC), thymus, lymph node (LN), spleen, bone marrow (BM), NK-cells, and brain from Biochain/BioCat (Heidelberg, Germany), and RNA from peripheral CD19-positive B-cells, CD3-positive T-cells, and CD34-positive stem cells from Miltenyi Biotec (Bergisch Gladbach, Germany). cDNA was synthesized from 5 μg RNA by random priming using Superscript II (Invitrogen). Real-time quantitative (RQ)-PCR analysis was performed with the 7500 Real-time System, using commercial buffer and primer sets (Thermo Fisher Scientific, Darmstadt, Germany). Quantification of MSX1 was performed as described previously [28]. For normalization of expression levels we analyzed the transcript of TATA box binding protein (TBP). We used the ddCT-method and the obtained values are indicated in relation to one sample which was set to 1.

For genomic copy number quantification of exon 1 from SMURF2 we extracted genomic DNA using the High Pure PCR Template Preparation Kit (Roche Diagnostics, Mannheim, Germany). The following oligonucleotides were used for Q-PCR analysis: SMURF2-1 5’-’CTCATGGCGACGACTCTCGGCAC-3’, SMURF2-2 5’-GGCGAGGCGCGGCGGAGTCACC-3. For normalization we used MEF2C as described previously [87].

Quantitative analyses were performed in triplicate. Standard deviations are presented in the figures as error bars. Statistical significance was assessed by *T*-Test and the calculated p-values indicated by asterisks (^*^p < 0.05, ^**^p < 0.01, ^***^p < 0.001, n.s. not significant).

### Protein analyses

Western blots were generated by the semi-dry method. Protein lysates from cell lines were prepared using SIGMAFast protease inhibitor cocktail (Sigma). Proteins were transferred onto nitrocellulose membranes (Bio-Rad, München, Germany) and blocked with 5% dry milk powder dissolved in phosphate-buffered-saline buffer (PBS). The following antibodies were used: alpha-tubulin (Sigma), NKX2-2 (Aviva Systems Biology, San Diego, CA, USA), ERK1 (Santa Cruz Biotechnology, Heidelberg, Germany), and phospho-ERK (Santa Cruz Biotechnology). For loading control blots were reversibly stained with Poinceau (Sigma) and detection of alpha-tubulin (TUBA) was performed thereafter. Secondary antibodies were linked to peroxidase for detection by Western-Lightning-ECL (Perkin Elmer, Waltham, MA, USA). Documentation was performed using the digital system ChemoStar Imager (INTAS, Göttingen, Germany).

## SUPPLEMENTARY MATERIALS FIGURES AND TABLES








