# Supplementary material for: NKL homeobox gene NKX2-2 is aberrantly expressed in Hodgkin lymphoma
Source: Oncotarget. 2018 Dec 25;9(101):37480–96. doi: 10.18632/oncotarget.26459 (PMC6331023; doi:10.18632/oncotarget.26459)
Supplement: Supplementary file 1 [file oncotarget-09-37480-s001.pdf]

NKL homeobox gene NKX2-2 is aberrantly expressed in Hodgkin lymphoma

SUPPLEMENTARY MATERIALS

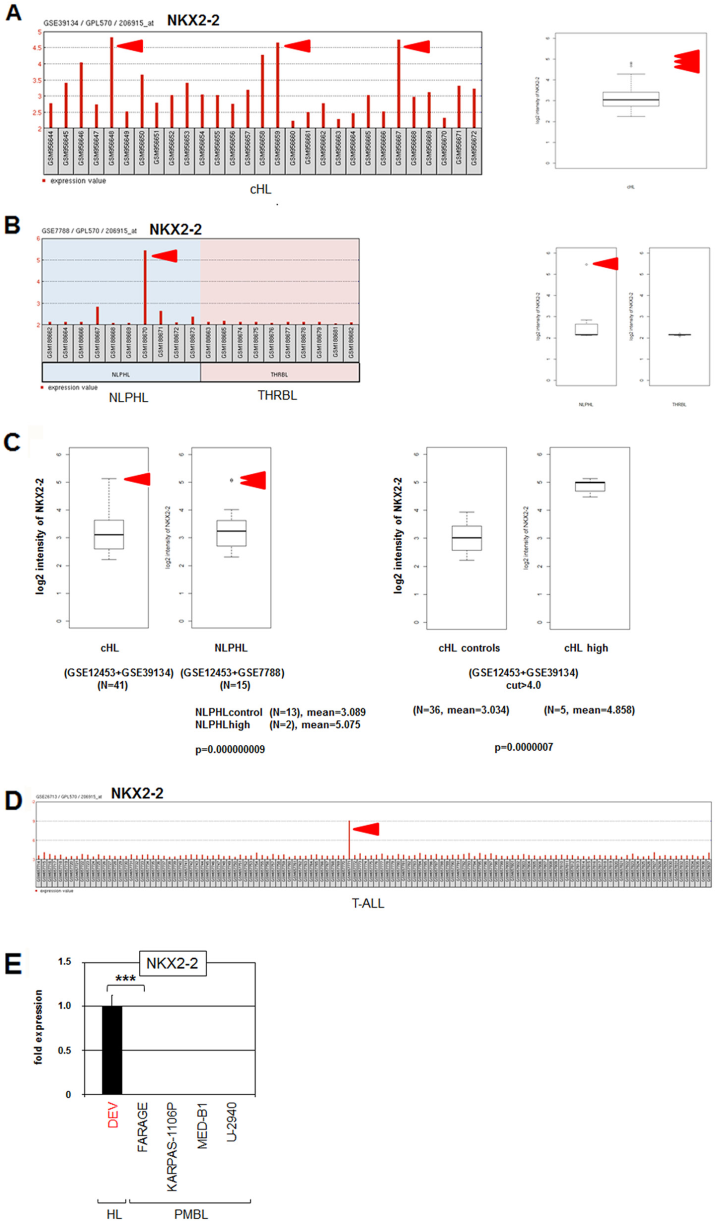

Supplementary Figure 1: Expression of NKX2-2 in HL and T-ALL patients and PMBL cell lines. **(A)** Expression profiling data of NKX2-2 (206915_at) in dataset GSE206915 containing samples of cHL patients are indicated as barplot (left) and boxplot (right). Red arrows indicate patients with elevated NKX2-2 expression levels. **(B)** Expression profiling data of NKX2-2 (206915_at) in dataset GSE7788 containing samples of NLPHL and T-cell/histiocyte rich B-cell lymphoma (THRBL) patients are indicated as barplot (left) and boxplot (right). **(C)** Analysis of combined datasets for NKX2-2 expression indicating elevated levels in cHL patients and two outliers of NLPHL patients (left, red arrow heads). These data demonstrate 2 of 15 (13%) of NLPHL patients significantly overexpressing NKX2-2. Splitting combined datasets GSE12453 and GSE39134 of cHL patients into enhanced NKX2-2 expressing patients (cHL high) and remaining controls (cHL controls) using a cut-off at 4.0, resulted in 5 of 41 (12%) of cHL patients significantly overexpressing NKX2-2 (right). **(D)** Expression profiling data of NKX2-2 (206915_at) in dataset GSE26713 containing 117 samples of T-ALL patients are indicated as barplot. **(E)** RQ-PCR analysis of NKX2-2 in four PMBL cell lines in comparison to HL cell line DEV.

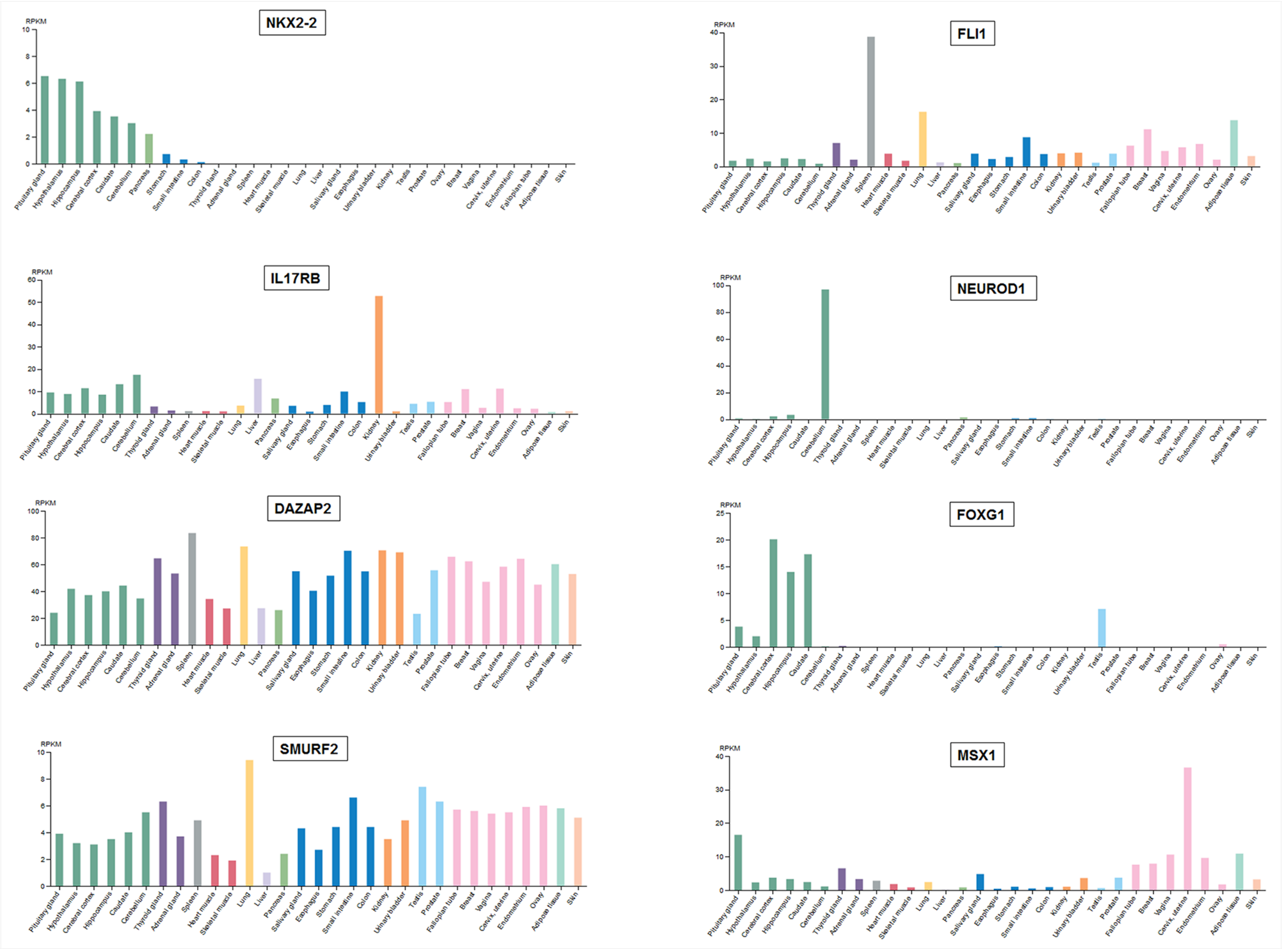

Supplementary Figure 2: RNA-seq data from the Human Protein Atlas. The Human Protein Atlas (https://www.proteinatlas.org/) was exploited for expression data of several genes including NKX2-2, IL17RB, DAZAP2, SMURF2, FLI1, NEUROD1, FOXG1 and MSX1. These diagrams indicate for several tissues the reads per kilobase million (RPKM), demonstrating particular gene activities. The tissue types are indicated by colors, e.g. brain in green.

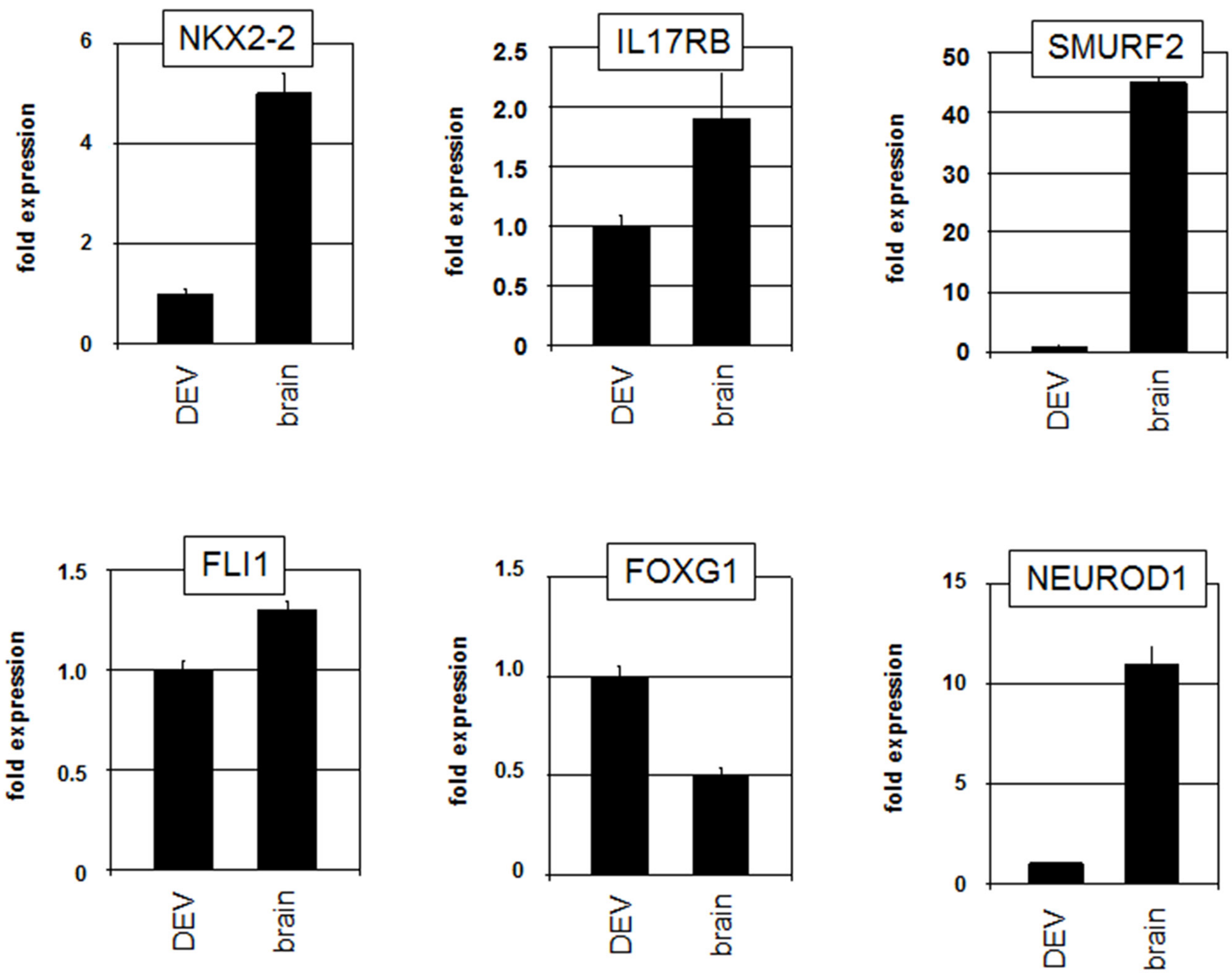

Supplementary Figure 3: Expression analyses in a human brain sample. RQ-PCR analysis of NKX2-2, IL17RB, SMURF2, FLI1, FOXG1 and NEUROD1 in DEV and a human brain sample. These data show similar gene activities in HL cell line DEV and in the primary brain sample.

NKX2-2 (p=1.36x10^{-4})

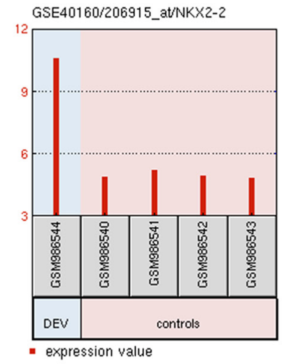

IL17RB (p=6.39x10^{-6})

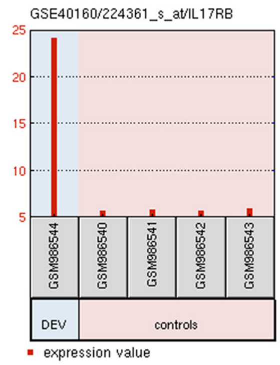

SYK (p=2.63x10^{-5})

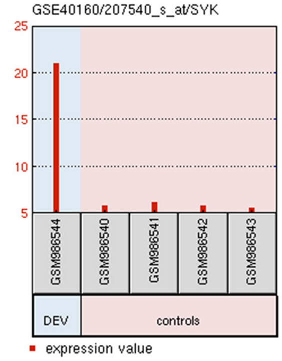

INPP5D (p=8.68x10^{-5})

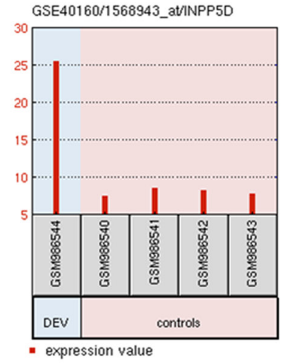

FOXG1 (p=2.49x10^{-7})

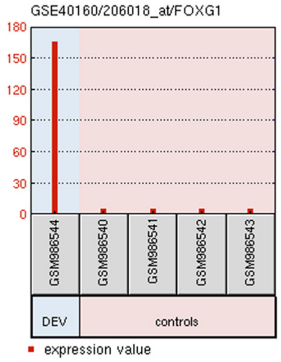

AUTS2 (p=6.20x10^{-5})

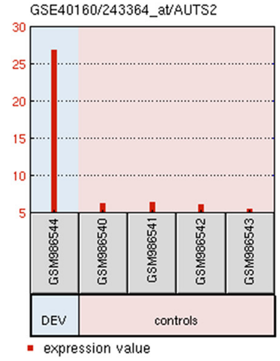

Supplementary Figure 4: Expression profiling data for HL cell lines. Dataset GSE40160 contains five HL cell lines including DEV. Expression data for NKX2-2, IL17RB, FOXG1 and AUTS2 were obtained by a GEO-supported comparative analysis showing 250 of the most significant differentially expressed genes. The calculated p-values are indicated.

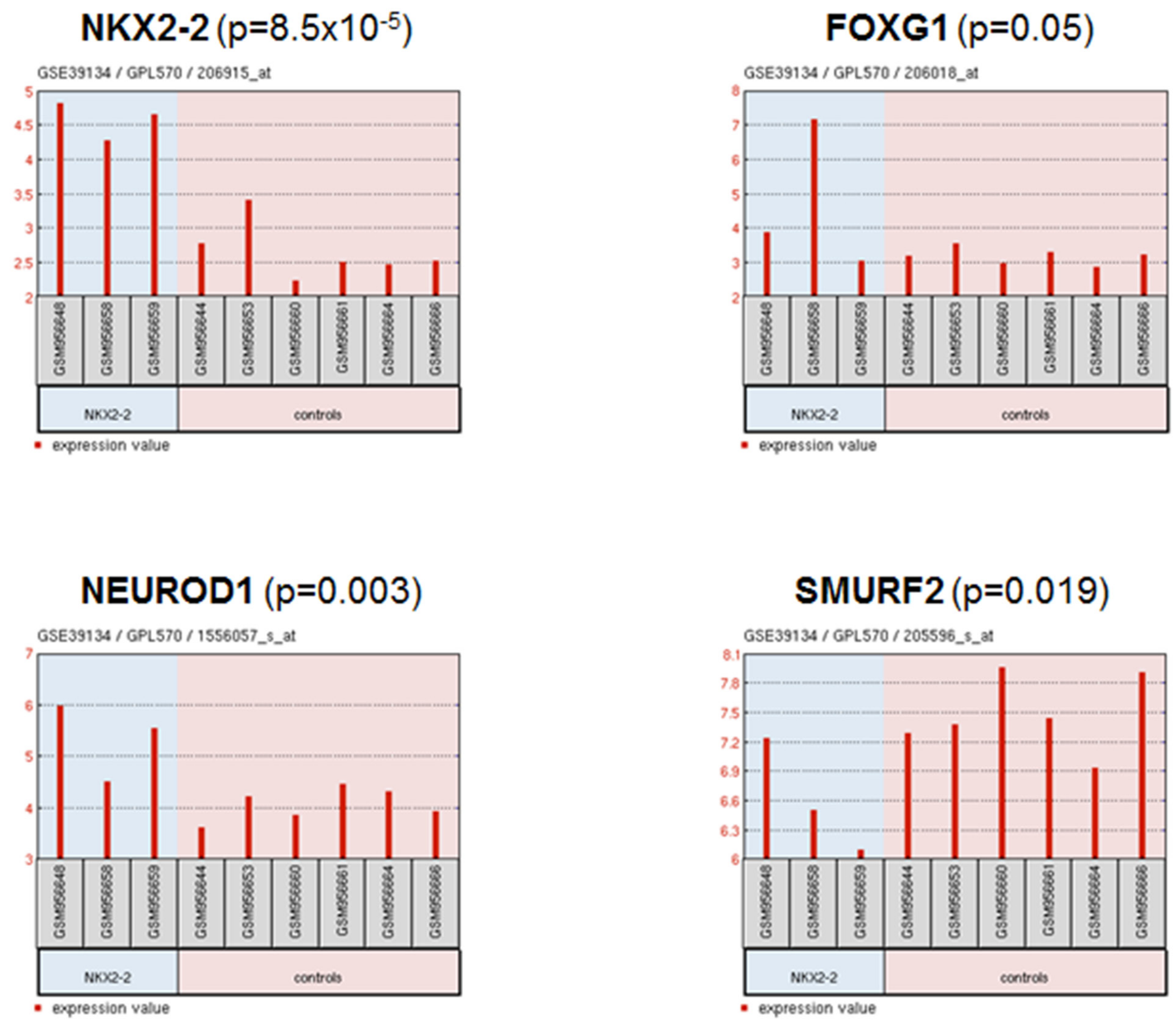

Supplementary Figure 5: Comparative expression profiling analysis in cHL. Using public dataset GSE39134 we compared selected three NKX2-2 positive cHL patients with six NKX2-2 negative cHL controls, identifying significant differences for NKX2-2, FOXG1, NEUROD1 and SMURF2.

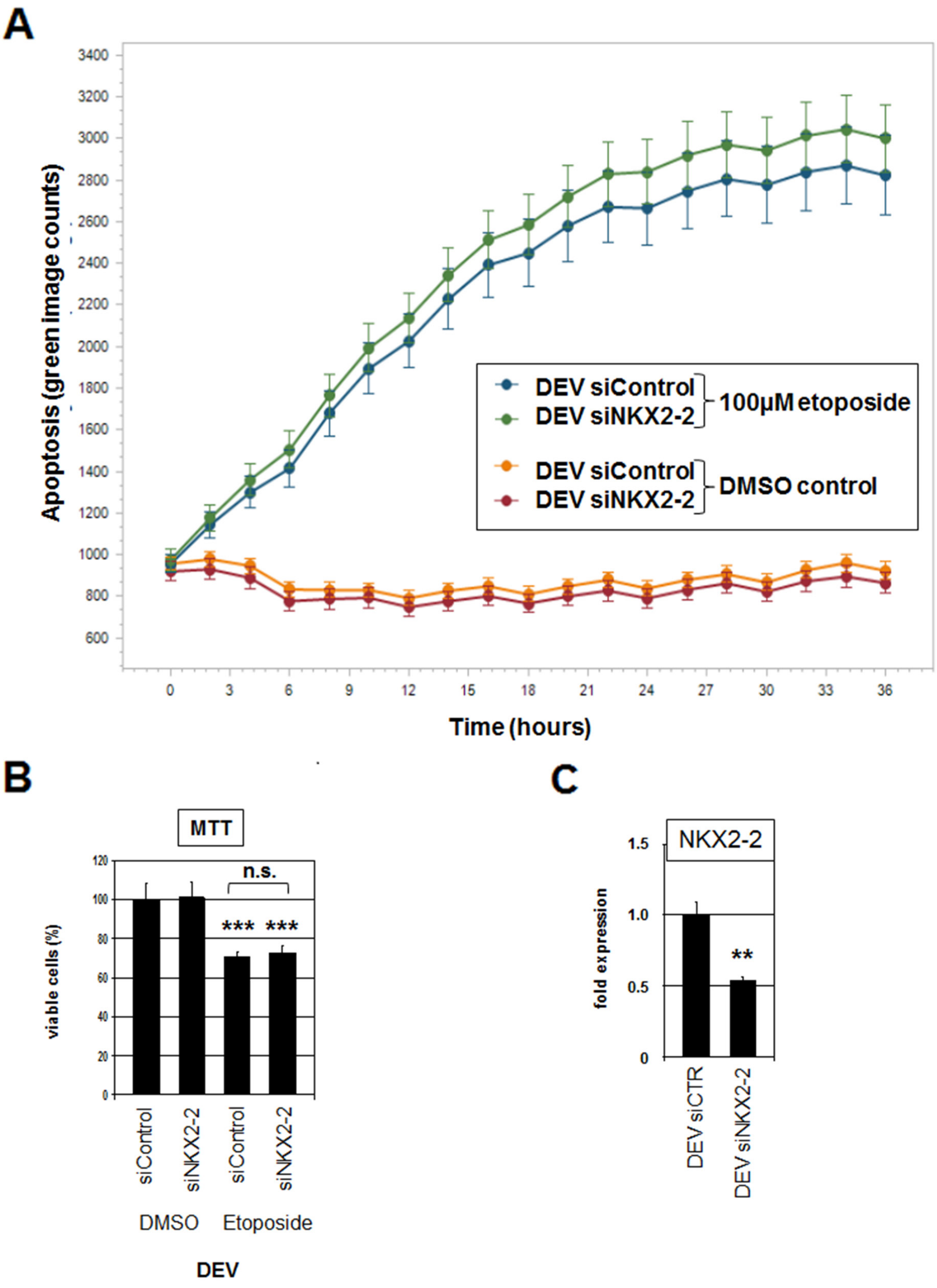

Supplementary Figure 6: Functional analysis of NKX2-2 in DEV. (A) DEV cells were treated by siRNA-mediated knockdown of NKX2-2 and subsequently incubated. Using an *in vivo* imaging system for DEV NKX2-2-knockdown cells with apoptosis-inducer etoposide (100 µM) showed no difference in comparison to the controls using an *in vivo* caspase-assay. **(B)** MTT assay was performed for DEV cells after the same treatment after 20h showing no difference as well. **(C)** The knockdown of NKX2-2 in DEV cells used for functional assays was confirmed by RQ-PCR.

Supplementary Table 1: Functional analysis of NKX2-2 in DEV. Dataset GSE40160 was analysed using an R-based online tool to reveal the top 250 most significant differentially expressed genes. Indicated are gene set IDs, calculated p-values, and names of the genes.

See Supplementary File 1

Supplementary Table 2: Functional analysis of NKX2-2 in cHL. Dataset GSE39134 was analysed using an R-based online tool to reveal the top 250 most significant differentially expressed genes. Indicated are gene set IDs, calculated p-values, and names of the genes.

See Supplementary File 2

Supplementary Table 3: Functional analysis of NKX2-2 in NLPHL. Dataset GSE7788 was analysed using an R-based online tool to reveal the top 250 most significant differentially expressed genes. Indicated are gene set IDs, calculated p-values, and names of the genes.

See Supplementary File 3
